# Supplementary figures and images for: Biomarker combination predicting imminent relapse after discontinuation of biological drugs in patients with rheumatoid arthritis in remission
Source: PLoS One. 2024 Mar 21;19(3):e0299450. doi: 10.1371/journal.pone.0299450 (PMC10956849; doi:10.1371/journal.pone.0299450)

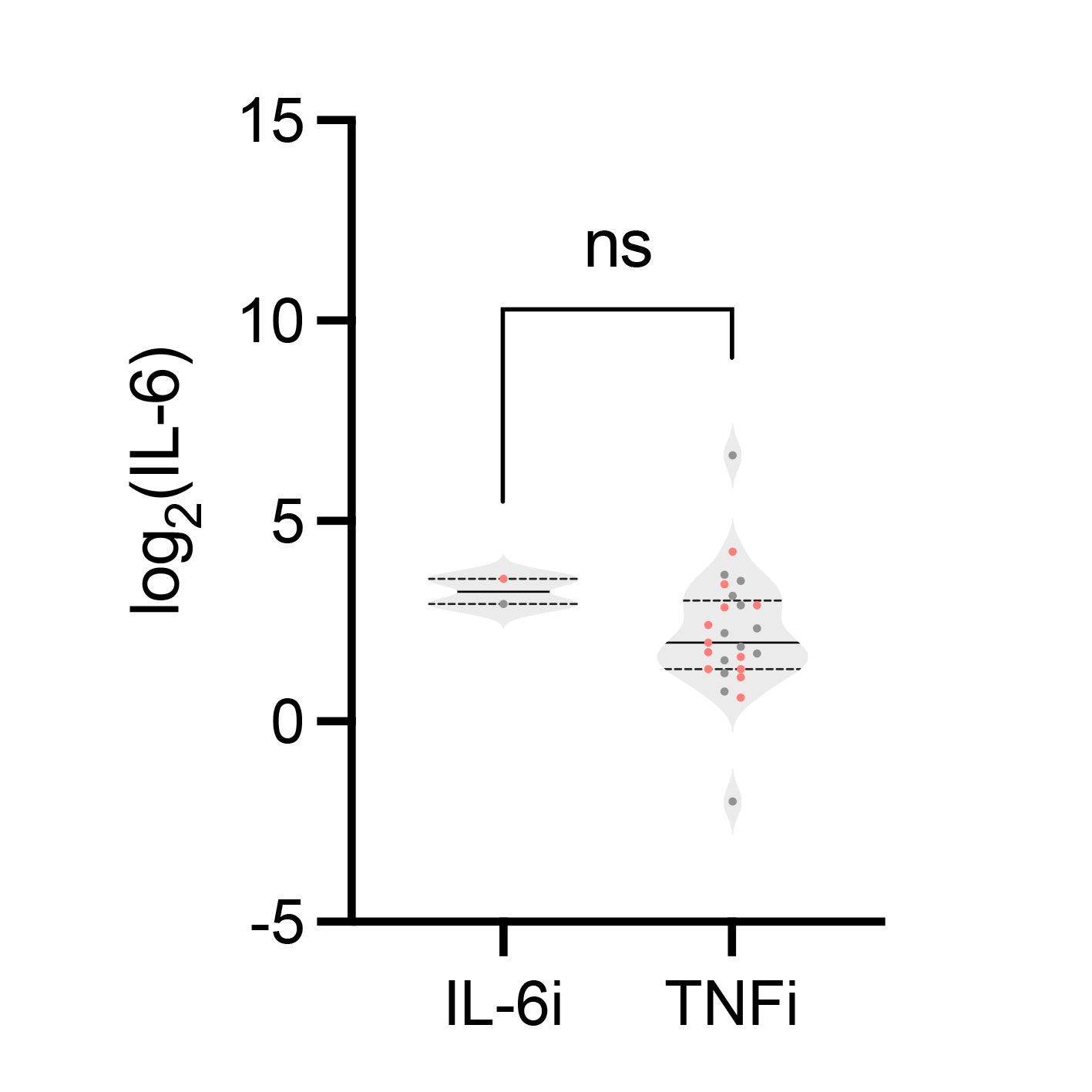

Supplement: S1 Fig — Log2-transformed IL-6 concentrations in patients immediately after bDMARD withdrawal. Orange-red and grey dots indicate patients of late relapse and non-relapse, respectively. Nonparametric Mann-Whitney test was used for the group comparison. Significance is defined by p < 0.05. ns, not significant. (TIF) [file pone.0299450.s001.tif]

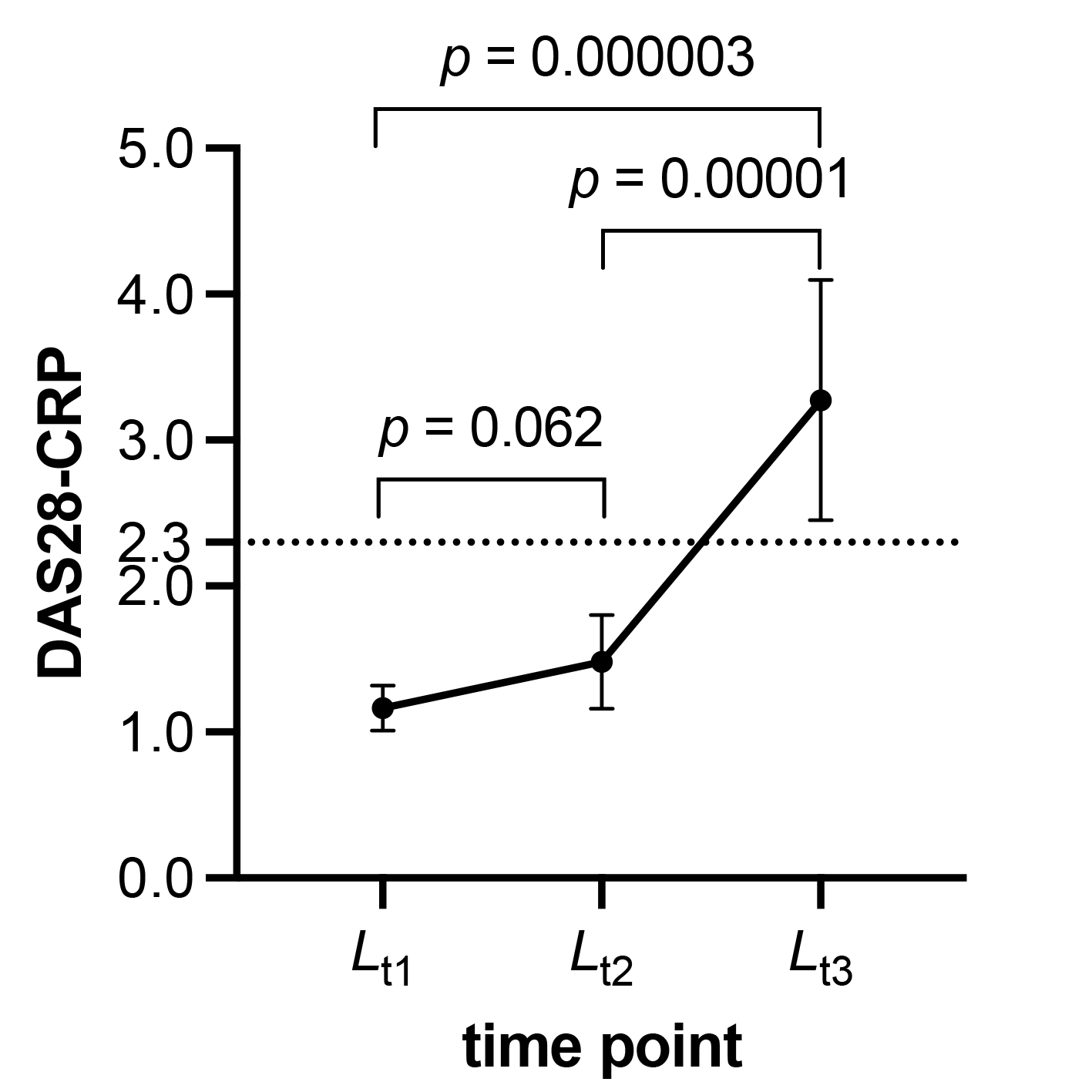

Supplement: S2 Fig — Paired t-test was employed for the group comparison, and the p-values were adjusted using the Bonferroni method. (TIF) [file pone.0299450.s002.tif]
