# Supplementary material for: Biomarker combination predicting imminent relapse after discontinuation of biological drugs in patients with rheumatoid arthritis in remission
Source: PLoS One. 2024 Mar 21;19(3):e0299450. doi: 10.1371/journal.pone.0299450 (PMC10956849; doi:10.1371/journal.pone.0299450)
Supplement: S1 Table — (PDF) [file pone.0299450.s003.pdf]

**S1 Table. Inflammatory and chemokine biomarkers measured.**

| Bio-Plex Pro human Inflammation 1 panel | | Bio-Plex Pro human chemokine panel | |
|---|---|---|---|
| APRIL/TNFSF13 | IL-27 (p28) | 6Ckine/CCL21 | IL-16 |
| BAFF/TNFSF13B | IL-28A/IFN-λ2 | BCA-1/CXCL13 | IP-10/CXCL10 |
| sCD30/TNFRSF8 | IL-29/IFN-λ1 | CTACK/CCL27 | I-TAC/CXCL11 |
| sCD163 | IL-32 | ENA-78/CXCL5 | MCP-1/CCL2 |
| Chitinase-3-like 1 | IL-34 | Eotaxin/CCL11 | MCP-2/CCL8 |
| gp130/sIL-6Rβ | IL-35 | Eotaxin-2/CCL24 | MCP-3/CCL7 |
| IFN-α2 | LIGHT/TNFSF14 | Eotaxin-3/CCL26 | MCP-4/CCL13 |
| IFN-β | MMP-1 | Fractalkine/CX3CL1 | MDC/CCL22 |
| *IFN-γ | MMP-2 | GCP-2/CXCL6 | MIF |
| *IL-2 | MMP-3 | GM-CSF | MIG/CXCL9 |
| sIL-6Rα | Osteocalcin | Gro-α/CXCL1 | MIP-1α/CCL3 |
| *IL-8 | Osteopontin | Gro-β/CXCL2 | MIP-1δ/CCL15 |
| *IL-10 | Pentraxin-3 | I-309/CCL1 | MIP-3α/CCL20 |
| IL-11 | sTNF-R1 | *IFN-ϒ | MIP-3β/CCL19 |
| IL-12 (p40) | sTNF-R2 | IL-1β | MPIF-1/CCL23 |
| IL-12 (p70) | TSLP | *IL-2 | SCYB16/CXCL16 |
| IL-19 | TWEAK/TNFSF12 | IL-4 | SDF-1α+β/CXCL12 |
| IL-20 | | IL-6 | TARC/CCL17 |
| IL-22 | | *IL-8/CXCL8 | TECK/CCL25 |
| IL-26 | | *IL-10 | TNF-α |

* IFN-γ, IL-2, IL-8, and IL-10 were measured in both panels.
